# Understanding X-ray-induced isomerisation in photoswitchable surfactant assemblies

**DOI:** 10.3762/bjoc.20.176

**Published:** 2024-08-14

**Authors:** Beatrice E Jones, Camille Blayo, Jake L Greenfield, Matthew J Fuchter, Nathan Cowieson, Rachel C Evans

**Affiliations:** 1 Department of Materials Science & Metallurgy, University of Cambridge, 27 Charles Babbage Road, CB3 0FS, United Kingdom; 2 Diamond Light Source, Harwell Science and Innovation Campus, Didcot, Oxfordshire, OX11 0DE, United Kingdom; 3 School of Chemistry, Trinity College Dublin, University of Dublin, College Green, Dublin 2, Ireland; 4 Department of Chemistry, Molecular Sciences Research Hub, White City Campus, Imperial College London, 82 Wood Lane, London, W12 7SL, United Kingdom; 5 Institut für Organische Chemie, Universität Würzburg, Am Hubland, 97074 Würzburg, Germany

**Keywords:** arylazopyrazole, azobenzene, micelle, photoswitch, X-ray

## Abstract

Dynamic, responsive materials can be built using photosurfactants (PS) that self-assemble into ordered nanostructures, such as micelles or liquid crystals. These PS contain photoswitchable groups, such as azobenzene (Azo) or, more recently, arylazopyrazoles (AAPs), which change shape and polarity on photoisomerisation between the *E* and *Z* states, thus changing the self-assembled structure. Small-angle X-ray scattering (SAXS) is a powerful technique to probe the morphology of PS and can be used to measure the mechanisms of structural changes using in-situ light irradiation with rapid, time-resolved data collection. However, X-ray irradiation has been shown previously to induce *Z-*to*-E* isomerisation of Azo-PS, which can lead to inaccuracies in the measured photostationary state. Here, we investigate the effect of light and X-ray irradiation on micelles formed from two different PS, containing either an Azo or AAP photoswitch using SAXS with in-situ light irradiation. The effect of X-ray irradiation on the *Z* isomer is shown to depend on the photoswitch, solvent, concentration and morphology. We use this to create guidelines for future X-ray experiments using photoswitchable molecules, which can aid more accurate understanding of these materials for application in solar energy storage, catalysis or controlled drug delivery.

## Introduction

The design of smart materials whose properties can be controlled using external stimuli is of significant interest for diverse applications spanning soft robotics [1], energy storage [2] and drug delivery [3]. Light is an ideal stimulus as it is non-invasive and can be administered selectively with high spatiotemporal control. To build responsive materials, surfactants are particularly attractive due to their ability to self-assemble into different morphologies depending on their molecular structure and chemical environment. To that end, photoswitchable chromophores can be incorporated into amphiphilic molecules to create photosurfactants (PS), whose molecular shape and polarity can be modified using light [4–6]. Of the PS studied, most use an azobenzene (Azo) photoswitch that undergoes *trans*-to-*cis* (*E*-to-*Z*) isomerization on irradiation with UV light, typically forming a photostationary state (PSS) that contains mostly *Z* isomers. This can be reversed using blue light or heat in a process that is stable over many cycles [7]. Isomerisation of azobenzene leads to a change in its conformation and polarity which, when combined into a surfactant molecule, modifies the resulting molecular geometry and hydrophilicity [6]. This, in turn, affects the interfacial and self-assembly properties of the PS [4–6]. The uniquely tuneable properties of these photoswitchable molecules have led to their successful application in areas such as DNA compaction [8], photorheological fluids [9–10] and micellar catalysis [11]. However, the potential scope for Azo in practice is limited by its incomplete photoswitching to the metastable isomer in the PSS and its rapid thermal reversion to the *E* isomer [12–13]. To tackle this, arylazopyrazoles (AAPs) have emerged as promising alternatives, where one of the phenyl rings of Azo is replaced by a pyrazole, which improves several aspects of their performance, including quantitative photoswitching between isomers and significantly enhanced thermal stability of the *Z* isomer [13–15]. This has led to recent reports on the integration of AAPs into surfactants to form systems with phototuneable interfacial [16–18] and self-assembly properties [19–20]. However, further understanding of factors which affect the isomerisation of these new surfactants, and the effect this has on their self-assembled structures, is still needed to tailor them towards application.

Small-angle scattering is a powerful technique that can be used to determine the structure and interactions of materials on length scales of 1–100s of nm. As a result, it has been used to study the changes in self-assembled morphologies of PS on irradiation with light [4,21–25]. In particular, the high brilliance of synchrotron X-ray sources enables the mechanisms of structural changes in PS to be studied, using in-situ light irradiation with time-resolved data collection. For example, Tribet and co-workers used this approach to explore the kinetics of micellisation and dissolution of cationic Azo-PS, both on their own and in mixed micelles with lipids, on irradiation with either UV or blue light [21–22]. In addition, Ober et al. showed that in-situ UV irradiation stimulates a steady decrease in bilayer thickness for vesicles formed using Azo-modified phosphatidylcholine lipids, due to the shorter lipid tail length in the *Z* isomer [26]. Notably, these authors observed that the X-rays themselves also induced *Z*–*E* isomerisation in Azo-lipids, which they attributed to the X-ray radiolysis of water, which produces radicals and reactive species that can catalyse *Z*–*E* conversion [27]. This effect was greater using low-energy X-rays (8 keV) due to their greater photoabsorption in water, thus leading the authors to conclude that higher energy (≈36 keV) should be used for future small-angle X-ray scattering (SAXS) experiments on photoswitchable systems. However, this may not always be achievable, depending on the X-ray energy available from laboratory or synchrotron sources. Moreover, this effect may have significant consequences when measuring any light-responsive materials using X-rays, from self-assembled PS systems (using SAXS) to crystalline or powder samples (using X-ray diffraction) [28–29]. In addition to this, damage to amphiphilic samples due to X-ray irradiation has been widely reported, leading to effects such as ionisation and structural reordering [30–31]. It follows that improved understanding of the effect of X-rays on photoswitchable surfactants is needed to design protocols that ensure the *Z*-rich PSS can be measured appropriately.

**Figure 1:**
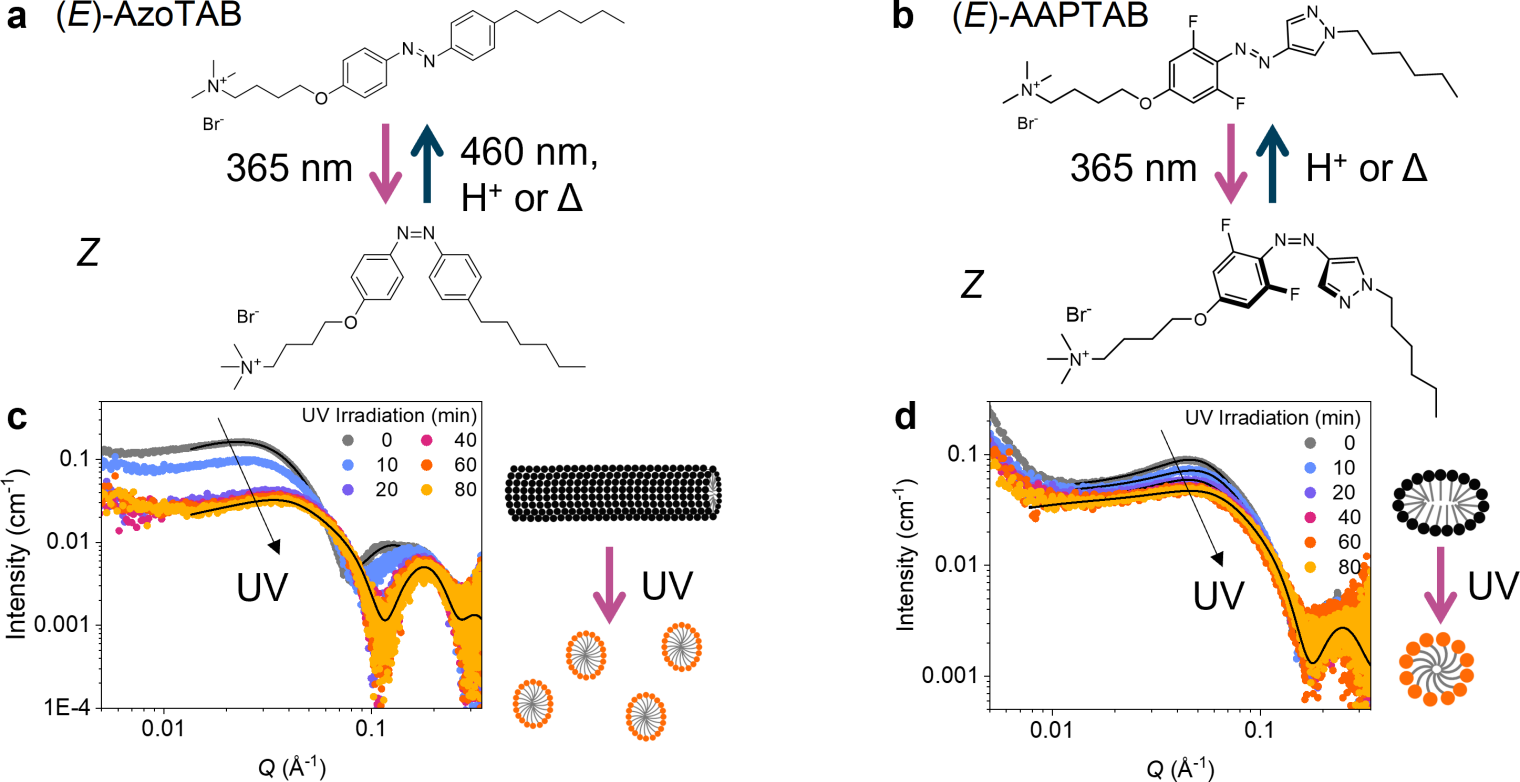
*E*–*Z* isomerisation of (a) AzoTAB and (b) AAPTAB under UV light (365 nm) results in a change in shape and polarity of the structures. This is reversible using blue light (460 nm, AzoTAB only), heat (Δ) or acid catalysis (both AzoTAB and AAPTAB). In-situ UV (365 nm) irradiation results in sequential change to the SAXS patterns for (c) AzoTAB and (d) AAPTAB (both 50 mM in water), attributed to changes in the micelle shape on *E*–*Z* isomerisation. Fits to the SAXS data (solid black lines) show micelle morphology changes from (c) cylindrical to ellipsoidal (AzoTAB) and (d) ellipsoidal to spherical (AAPTAB), which are depicted in the schematic representations. Figure 1c was modified from [33] (**©** 2024 B. E. Jones et al., published by Journal of Synchrotron Radiation, distributed under the terms of the Creative Commons Attribution 4.0 International License).

To address this, here we investigate the effects of light- and X-ray irradiation on PS assemblies to further understand the parameters which influence X-ray-induced *Z*–*E* isomerisation. Two different cationic PS molecules are studied, based on the ubiquitous cetyltrimethylammonium bromide (CTAB), which contain either an Azo (i.e., AzoTAB, Figure 1a) or arylazopyrazole (i.e., AAPTAB, Figure 1b) photoswitch. AzoTAB and AAPTAB were chosen as they display distinct changes in micelle morphology on irradiation with UV light to form the *Z*-rich PSS [4,19]. In addition to this, AAPTAB has a high thermal half-life at room temperature (5.7 years [19]), meaning there is no significant contribution from thermal *Z*–*E* isomerisation over the course of these experiments. Using in-situ UV- and visible-light irradiation with SAXS, here we measure the intermediate structures formed during photoisomerisation of these PS for the first time. Once isomerised to the *Z*-rich PSS, the effect of X-ray irradiation is studied using time-resolved SAXS collection, where the photoswitch, solvent and concentration are all shown to impact the rate and extent of structural change. With comparison to the rate of *Z*–*E* isomerisation on addition of acid to the PS systems, we show that factors beyond the production of protons (H^+^) upon X-ray radiolysis of water may have an effect to produce the large, rapid structural changes observed using SAXS. To conclude, we create a set of guidelines for X-ray experiments using photoswitchable molecules, which is required to ensure these systems are understood accurately on designing them for applications such as solar energy storage [32], catalysis [11] or drug delivery [3].

## Results and Discussion

### Effect of light irradiation

First, in-situ UV irradiation with SAXS was used to determine the mechanisms for morphology changes in AzoTAB and AAPTAB systems on *Z*–*E* isomerisation. A concentration of 50 mM in water was chosen for these experiments, as this is well above the critical micelle concentration (CMC) for both surfactants (CMC in water for (*E*)-AzoTAB = 0.2 mM [4], (*E*)-AAPTAB = 5.4 mM [20]), and previous SAXS measurements have shown morphology changes between micelles formed for the *E* and *Z* isomers at or near this concentration [4,19]. For AzoTAB as the native, *E*-isomer, the SAXS pattern is characteristic of interacting micelles and can be fitted to an ellipsoidal cylindrical shell model (Figure 1c), consistent with previous results [4,33]. A Hayter–Penfold structure factor was incorporated into the fits to account for screened Coulombic repulsion between the charged micelles [34]. In-situ UV (365 nm) irradiation triggers a sequential change in the SAXS profile from the AzoTAB (Figure 1c), resulting in a steady decrease in the scattering intensity and a shift in the Guinier plateau to higher *Q* values. These changes are visible within 10 minutes of UV irradiation and stabilise with no further structural changes after around 20 minutes. It should be noted that a lag between the time taken to reach the PSS and the time for the new micelle morphology to form is expected. This is due to the additional time that is required for diffusion and reassembly of the AzoTAB in the *Z* conformation into the new equilibrium structure [22]. The scattering curve for the UV-induced PSS fits to an ellipsoidal core-shell micelle model (see Figure 1c and Table S1, Supporting Information File 1 for full fit details), which indicates that the initial cylindrical micelles break up into smaller fragments along their length on irradiation, resulting in micelles with a higher spontaneous curvature [33]. Previously, samples of lower concentration (20 mM, still > CMC) showed no change from the cylindrical morphology on isomerisation [4]. Here, we propose that the shorter cylinder length, due to the higher concentration, could result in the structure being closer to the cylindrical-ellipsoidal morphology boundary. Furthermore, a tendency to form higher-curvature morphologies on *E*–*Z* isomerisation has been seen for various Azo-PS, both with shorter and longer alkyl chain tails and spacer groups in comparison to the AzoTAB structure studied here. This can be explained by an increased tail-group volume of the *Z* isomer, which favours a higher spontaneous curvature in the amphiphile packing [4].

Similarly, the SAXS signal for AAPTAB shows a sequential change on UV irradiation (Figure 1d), reaching an equilibrium state after 40 minutes. Model fitting indicates that this is a result of an ellipsoid-to-sphere morphology change in the AAPTAB micelles, which matches reports from previous work using ex-situ irradiation [19]. The dimensions of the micelles change from a polar and equatorial radius of 24 and 13 Å to a single spherical radius of 18 Å (Table S2, Supporting Information File 1). This morphology change can be attributed to two changes in the AAPTAB on *E*–*Z* isomerisation [19]. Firstly, the shape change of the AAPTAB to the bent, “T-shape” conformation prevents the π–π stacking which was previously possible in the more planar, *E* isomers that were arranged in elongated, ellipsoidal micelles. Secondly, the change in geometry and polarity of the AAP photoswitch on isomerisation to the *Z* state [35] results in an effective incorporation of the AAP group into the hydrophilic headgroup of the PS. This means that the hydrophobic tail-group becomes just the pendant hexyl chain. This acts to increase the headgroup area of the surfactant and the spontaneous curvature of the resulting self-assembled micelles [4]. In-situ UV irradiation shows that *E*–*Z* isomerisation results in changes to the morphology of AzoTAB and AAPTAB micelles, where intermediate structures are formed, which could be due to the presence of a mixed state of *E* and *Z* isomers or gradual equilibration of the system into the new morphologies adopted by the *Z* isomer.

### Effect of X-ray irradiation

**Figure 2:**
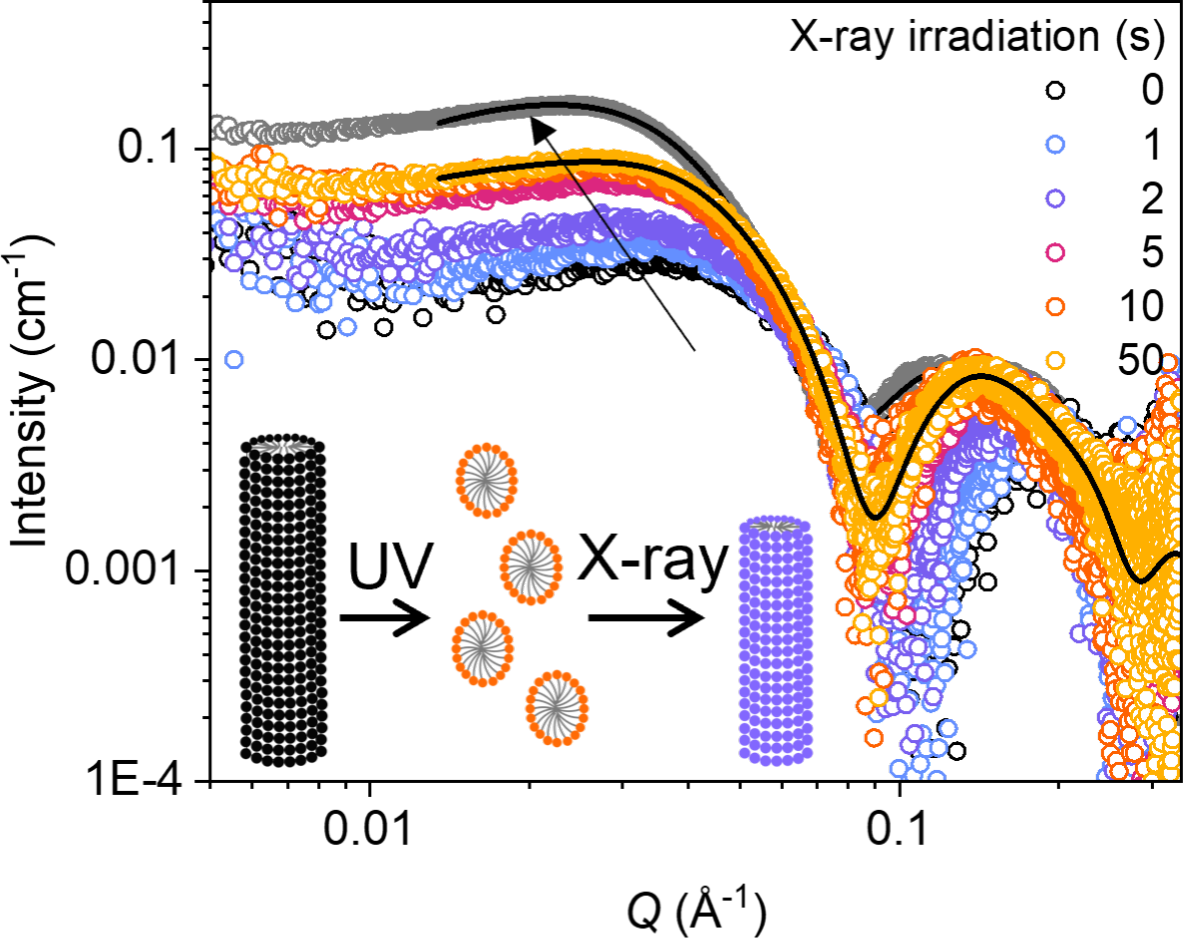
SAXS curves for AzoTAB (50 mM in water) showing the transition from the *Z*-rich PSS to the *E*-rich state with increasing X-ray exposure time. Fits to the data (black lines) indicate a morphology change from ellipsoidal to cylindrical micelles, as shown in the schematic insert (to scale). The grey circles indicate the original *E*-isomeric state before UV irradiation. The graphic is modified from [33] (© 2024 B. E. Jones et al., published by Journal of Synchrotron Radiation, distributed under the terms of the Creative Commons Attribution 4.0 International License).

Next, the effect of X-ray irradiation on the *Z*-rich PSS was investigated. AzoTAB (50 mM in water) was first irradiated with UV light in-situ to reach the PSS (80 min). The subsequent effect of X-ray irradiation (13 keV) was tracked over a total time of 50 s, taking individual exposures of 500 ms, separated by a delay-time of 100 ms. Irradiation with X-rays results in a partial return of the scattering pattern to that of the native, *E* isomer (Figure 2). Changes occur immediately, after 1 s of X-ray exposure, and saturate after ca. 5 s. The changes are comparable to those observed on *Z*–*E* isomerisation induced using blue (460 nm) light or heating to 55 °C but occur at a much faster rate (Figure S2, Supporting Information File 1). Fits to the data show a return to the cylindrical micelle morphology present in the *E* isomer, but with smaller dimensions of 98 Å length (cf. 136 Å in the *E* isomer), and radii of 13 and 15 Å in the polar and equatorial directions (Table 1). A similar reduction in micelle size was seen on reverse isomerisation using blue light (Table S1, Supporting Information File 1), which may suggest that the larger micelle size in the *E* isomer is obtained by slow agglomeration over time.

**Table 1:** Micelle morphologies and dimensions for PS obtained from SAXS data fits after UV (365 nm) and X-ray irradiation.

PS	irradiation time		morphology	*r*_p_^a^ (Å)	*r*_eq_^b^ (Å)	*l*^c^ (Å)
	UV (min)	X-ray (s)				

AzoTAB	0	0	cylinder	31.4 ± 0.1	11.5 ± 0.1	136.0 ± 0.2
	80	0	ellipsoid	13.3 ± 0.1	19.3 ± 0.2	—
	80	50	cylinder	12.7 ± 0.1	14.7 ± 0.1	97.5 ± 0.6
AAPTAB	0	0	ellipsoid	23.8 ± 0.1	13.4 ± 0.1	—
	80	0	sphere	18.4 ± 0.1		—
	80	50	ellipsoid	23.8 ± 0.2	13.4 ± 0.1	—

^a^Polar radius. ^b^Equatorial radius. ^c^Length of cylindrical micelle.

The interaction of ionising radiation, such as X-rays, with water is known to result in a radiolysis process that generates a wide variety of primary species, including e^−^, HO^•^, H^•^, HO_2_^•^_,_ H^+^, OH^−^, H_2_O_2_ and H_2_ [36]. For Azo photoswitches, the formation of H^+^ species, i.e., the acidification effect, can catalyse the *Z*–*E* isomerisation reaction. This is due to protonation of one of the nitrogen atoms in the azo bond (N=N), resulting in a decrease in the double bond character and a lowering of the energy barrier to isomerisation [37–39]. Additionally, *Z*–*E* isomerisation can be catalysed electrochemically in the presence of free electrons [40] or holes [27]. The wide variety of radical and charged species produced on water radiolysis may therefore play a complicated role in inducing *Z*–*E* isomerisation via a number of different mechanisms. No matter the mechanism, the morphology changes on X-ray irradiation appear consistent with the hypothesis that the *E* isomer is reformed, as shown by the return of the micelle shape of this isomer. The fast rate of the morphology change, in comparison to using blue light or heating to 55 °C, shows rapid rates of *Z*–*E* isomerisation from X-ray-induced catalysis, which may be due to the presence of multiple different catalysing species and therefore isomerisation pathways. In contrast, there was negligible change in the SAXS pattern for AzoTAB in the native, *E* isomer over this irradiation time (Figure S4, Supporting Information File 1). This supports the hypothesis that the changes occur due to *Z*–*E* isomerisation, rather than X-ray-induced ionisation or beam damage to the AzoTAB, as seen in similar amphiphilic systems [30–31]. In the case of ionisation, both stereoisomers would be expected to be affected equally.

**Figure 3:**
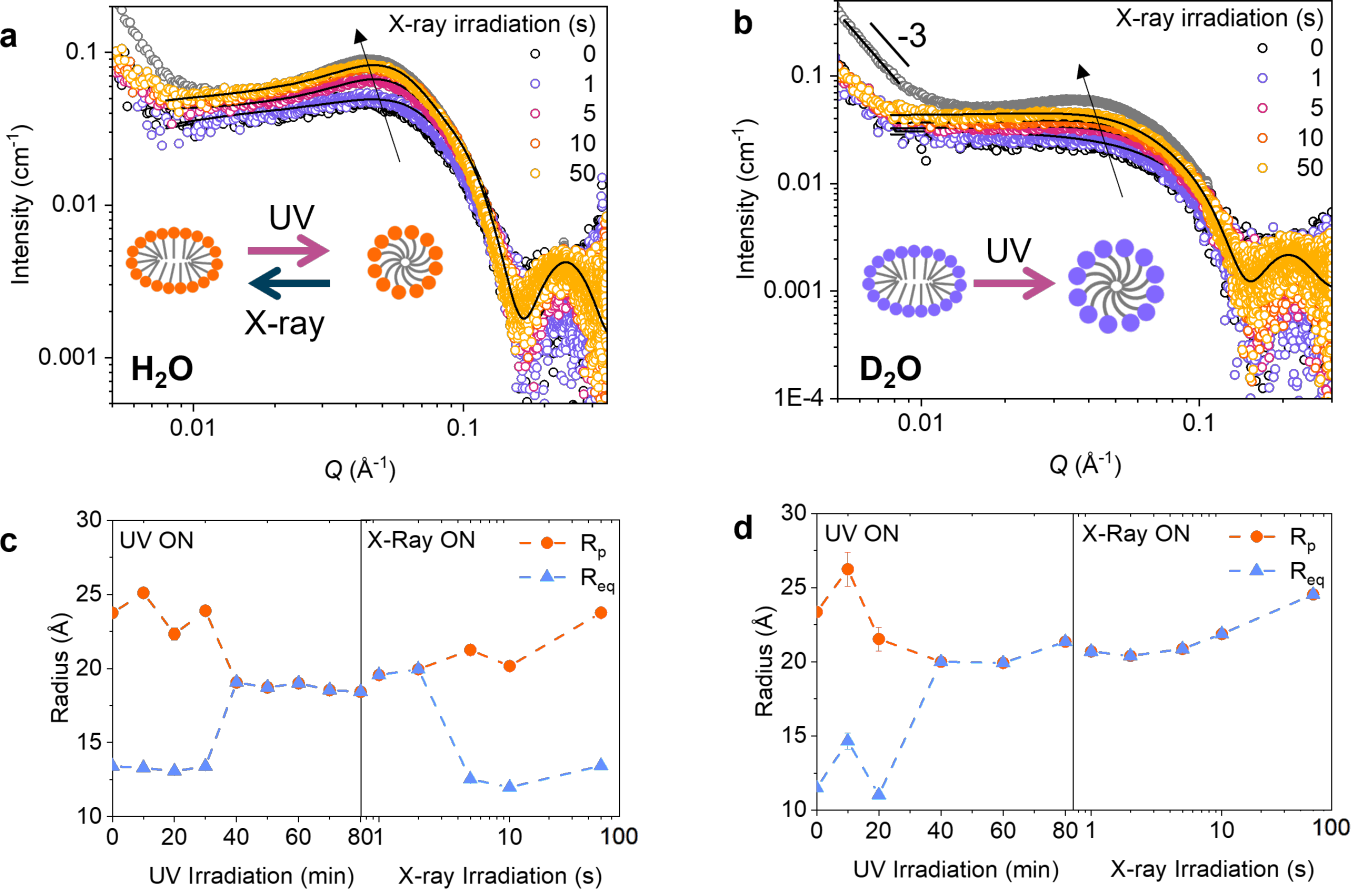
SAXS curves for the *Z*-rich PSS of AAPTAB (50 mM) in (a) water (H_2_O) and (b) deuterium dioxide (D_2_O) show changes to the micelle shape and size on X-ray exposure. Fits to the data (black lines) indicate that a spherical-to-ellipsoidal transition occurs in H_2_O but not in D_2_O. Note that the grey circles indicate the native, *E*-isomer state, and the slope of −3 at low *Q* values (b) indicates the presence of larger-scale aggregation. The dimensional changes to the ellipsoidal and spherical micelles are shown for (c) H_2_O and (d) D_2_O, where *R*_p_ is the polar radius and *R*_eq_ is the equatorial radius.

In comparison to Azo, the AAP moiety is much more stable as the *Z* isomer, with a thermal half-life of 5.7 years at room temperature [13]. This means that *Z*–*E* isomerisation cannot be easily induced using gentle heating or visible light, as shown by the stability of the UV–vis absorbance spectra and SAXS patterns under these conditions (Figures S1 and S3, Supporting Information File 1). In contrast, irradiation with X-rays leads to significant changes in the SAXS patterns due to induced *Z*–*E* isomerisation (Figure 3a). Model fitting to the SAXS data shows that X-ray irradiation induces a sequential increase in the radius of the spherical micelles from 0.5 to 2 s, and a return to the ellipsoidal morphology after 5 s (Figure 3c). After 50 s, the micelle dimensions match those obtained for the AAPTAB in the *E*-isomeric form (Table 1 and Table S2, Supporting Information File 1). As with the AzoTAB sample, there is little change to the SAXS profile for AAPTAB in the native, *E* isomer after the same X-ray irradiation time (Figure S4, Supporting Information File 1), suggesting that the X-ray irradiation alone does not cause AAPTAB ionisation or affect the micelle morphology. As with Azo, *Z*–*E* isomerisation in AAP photoswitches can be catalysed efficiently using oxidising or reducing species [41]. This means that, despite the increased thermal stability of the *Z* isomer, AAP photoswitches are also susceptible to *Z*–*E* isomerisation on X-ray irradiation due to the presence of catalysing ionic and radical species from radiolysis of the surrounding water. This can be seen by a partial return of the UV–vis absorbance spectrum of AAPTAB from the *Z* to the *E* isomer after X-ray irradiation (Figure S7, Supporting Information File 1).

To further investigate the mechanism for *Z*–*E* isomerisation using X-rays and associated structural changes, we conducted an identical experiment using D_2_O instead of H_2_O. The change of solvent leads to an increase in the viscosity, from 0.89 in H_2_O to 1.10 mPa⋅s in D_2_O [42], which is attributed to slow diffusion of micelles within the medium. Furthermore, the rate of H^+^/D^+^ exchange between the solvent and the photoswitch will decrease due to the heavier deuterium atoms in D_2_O, resulting in an effective weakening of the acidification effect from radiolytic D^+^ formation [43]. The change from H_2_O to D_2_O may also affect the surfactant self-assembly due to the change in hydrogen-bond strength [44]. Fitting to the SAXS data shows that AzoTAB and AAPTAB in the *E*-isomeric forms form elliptical cylindrical and ellipsoidal micelles, respectively, with comparable dimensions to those in H_2_O (Tables S1 and S3, Supporting Information File 1). However, there is a large difference in the interactions between AAPTAB micelles on switching solvents, which can be seen as a decrease in the interaction hump in the SAXS pattern at *Q* ≈ 0.045 Å^−1^ (Figure 3a and Figure 3b). This may be due to a decrease in the CMC, as expected for TAB surfactants in D_2_O in comparison to H_2_O [45], which drives the formation of larger-scale aggregates within the solution. This is visible as a strong power-law decay in the low-Q region of the SAXS pattern, where *I*(*Q*) ∝ *Q^−^*^3^ (Figure 3b), and could result in fewer micelles in solution and fewer interactions between them. Despite this, both AzoTAB and AAPTAB undergo similar micelle morphology transitions on UV irradiation in D_2_O as compared to those in H_2_O (cylindrical-to-ellipsoidal and ellipsoidal-to-spherical, respectively, Tables S1 and S3, Supporting Information File 1).

On subsequent X-ray irradiation of the *Z*-rich PSS, AzoTAB in D_2_O shows partial recovery of the elliptical cylindrical micelle shape present in the *E* isomer (Table S1, Supporting Information File 1); however, a longer exposure time is needed to induce changes compared to those occurring in H_2_O (10 s cf. 5 s). The effect is much more pronounced for the AAPTAB system, where there is no evidence of the micelle morphology changing back to the *E* form on X-ray irradiation in D_2_O.The fits instead show a slow increase in the radius of the spherical micelles, from 20 to 25 Å after 50 s of irradiation (Figure 3d), but the morphology transition to the ellipsoidal shape is not complete. This could be due to both the slower rate of D^+^ exchange with the photoisomers, leading to a slower rate of *Z*–*E* isomerisation via this mechanism. Furthermore, the decreased rate of diffusion in the more viscous D_2_O could lead to slower rearrangement after reverse isomerisation into the *E* isomer morphology. This demonstrates that the solvent plays a crucial role in X-ray-induced reverse isomerisation in these systems, not only due to catalysis from the radiolysis effect, but also as the medium for structural rearrangements.

### Effect of acidification

**Figure 4:**
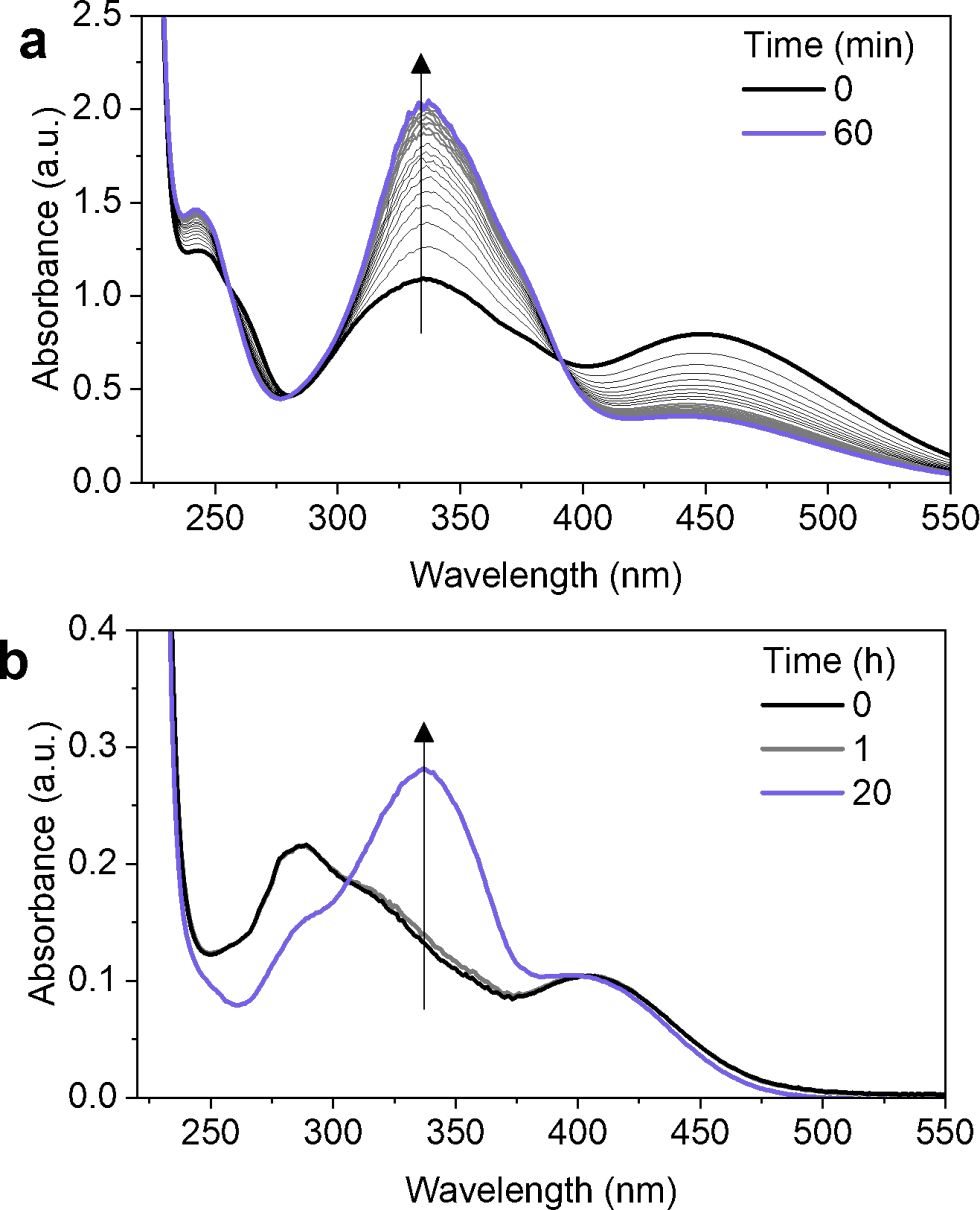
Addition of excess acid (pH = 0.4) induces *Z*–*E* isomerisation in AzoTAB and AAPTAB. UV–vis absorbance spectra for (a) AzoTAB (25 μM in water) and (b) AAPTAB (100 μM in water) after UV irradiation to form the *Z*-rich PSS and addition of excess hydrobromic acid as a function of time held in the dark. The grey lines indicate time intervals of (a) 3 minutes and (b) 1 hour.

The effect of acidification on isomerisation was further investigated using UV–vis absorbance spectroscopy, where excess hydrobromic acid (HBr) was added to AzoTAB and AAPTAB samples (25 μM) that had been preirradiated with UV light into the *Z*-rich PSS. HBr was selected as the acid to eliminate any effects from the Br^−^ counterion on the self-assembly behaviour [36]. AzoTAB (in the *Z*-rich PSS) showed a rapid response to acidification with near-complete reversal to the *E* isomer within 60 minutes (Figure 4a). In contrast, for AAPTAB, the process was much slower, with minimal change over 1 hour, but near-complete transition to the *E* isomer after 20 hours (Figure 4b). The excess acid added in these experiments generates a pH value of 0.4 in the samples. In comparison, the calculated pH change expected due to the X-ray radiolysis of water (see section 6, Supporting Information File 1) was found to be from pH 7 to ≈ pH 5–6 for the X-ray irradiation doses used in these experiments. Interestingly, both AzoTAB and AAPTAB show little difference in their time-dependent absorbance spectra from the *Z*-rich PSS over the course of 200 minutes at pH 5, 6 and 7 (Figures S8 and S9, Supporting Information File 1). This suggests that the X-ray-induced formation of H^+^ ions alone may not be strong enough to induce *Z*–*E* isomerisation on the millisecond timescales on which structural changes were observed using SAXS. We note that the temperature change due to heating effects from the X-ray beam is <0.4 °C for a single 500 ms frame (see section 7, Supporting Information File 1), meaning that this will not affect the *Z*–*E* isomer ratio. The diverse range of reactive radical species formed in the radiolysis process must therefore play a key role in catalysing the *Z*–*E* isomerisation via multiple mechanisms.

### Effect of solvent concentration

**Figure 5:**
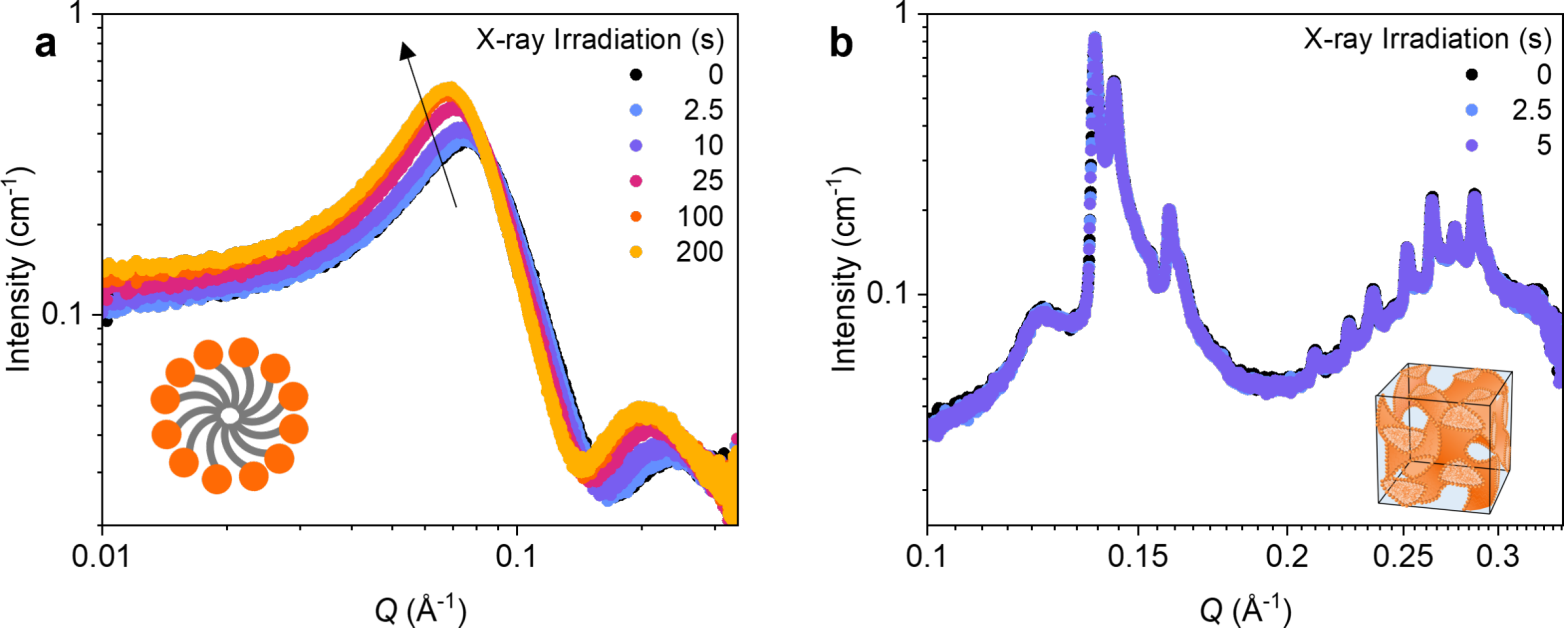
Effect of X-ray exposure time on high-concentration samples of AAPTAB in water, (a) 10 wt % and (b) 90 wt %, after ex-situ UV irradiation to form the *Z*-rich PSS. The schematic inserts illustrate the morphologies for the (a) isotropic micellar and (b) inverse bicontinuous gyroid cubic phases. The graphic depicted in Figure 5b was reproduced from [20] (© 2024 B. E. Jones et al., published by American Chemical Society, distributed under the terms of the Creative Commons Attribution 4.0 International License).

The solvent clearly plays a crucial role in enabling *Z*–*E* isomerisation of AzoTAB and AAPTAB on X-ray irradiation. To investigate this further, we decreased the concentration of solvent greatly to study two samples of AAPTAB at 10 and 90 wt % with respect to water. For such high surfactant loadings, additional factors must be considered such as increased absorbance and lower light penetration, as well as reduced diffusion due to higher viscosities. To counteract this, we chose to irradiate the samples ex-situ for 3.5 hours to achieve a *Z*-rich PSS, where the percentage isomerised has been determined previously using ^1^H NMR spectroscopy [20]. The 10 wt % sample was measured over 800 X-ray exposures of 250 ms each. The initial *Z*-rich PSS SAXS pattern shows a strong interaction peak at *Q* ≈ 0.08 Å, characteristic of an isotropic micellar mesophase with densely packed, strongly interacting micelles (Figure 5a). On X-ray irradiation, the interaction peak increases in intensity and shifts to lower *Q* values. This suggests that there is an increase in the micelle size on irradiation, which agrees with the previous results that the *E* isomer forms larger micelles than the *Z* isomer. The shift in the SAXS pattern was visible within 2.5 s of X-ray irradiation, with significant changes resulting after 200 s of irradiation. For AAPTAB at 90 wt % in water, a number of sharp, Bragg peaks are visible in the SAXS pattern (Figure 5b), which are characteristic of the long-range order present in a lyotropic liquid crystal (LLC) mesophase that can form from the self-assembly of surfactants at high concentrations in a solvent. An inverse bicontinuous gyroid cubic mesophase can be assigned using the *Q* positions of the Bragg peaks, which are in a characteristic ratio of √6:√8:√14:√16:√20:√22 [20]. The sample was measured over 20 exposures of 250 ms each, with little evidence of a change to the LLC order over this period. The lower rate of *Z*–*E* isomerisation on X-ray irradiation can be explained by several different factors. The lower free volume in the ordered, LLC structure could prevent rapid *Z*–*E* isomerisation on X-ray irradiation, despite the formation of ions and radical species which would catalyse the reaction. Alternatively, the lower volume of water within the sample (only 10 wt %) would mean that there is less X-ray radiolysis, fewer catalysing species produced and therefore a lower effect on the *Z*–*E* reaction. Interestingly, it has been shown previously that the efficiency of electron or hole catalysis of *Z*–*E* isomerisation increases with increasing photoswitch concentration, due to the closer proximity leading to more efficient transfer of the catalysing species between molecules [40]. This suggests that there is a breakdown in the efficiency of the catalytic transfer at the concentrations studied here, or there is a critical water concentration needed to produce a sufficient number of species to catalyse isomerisation. X-ray-induced *Z*–*E* isomerisation therefore has a prevalent effect up to solvent concentrations of 90 wt % water; however, the effects are significantly slowed on formation of dense, LLC phases with low solvent concentrations (10 wt %).

## Conclusion

In summary, with in-situ UV irradiation both AzoTAB and AAPTAB undergo morphological transitions due to *E*–*Z* isomerisation, producing micelles of higher spontaneous curvature. On subsequent X-ray irradiation both AzoTAB and AAPTAB in the *Z*-rich PSS revert to the micelle morphologies formed by the initial *E* isomers. It is thought that this is due to X-ray-induced radiolysis of water, which produces many primary species, including H^+^ ions, that are known to catalyse the *Z*–*E* isomerisation reaction. By switching the H_2_O solvent to D_2_O, we observed that the increased viscosity and reduced acidification acted to slow the reversal process, especially for the AAPTAB, supporting this hypothesis. However, investigations into the degree of isomerisation at different pH values for AzoTAB and AAPTAB in water suggest that the pH change caused by X-ray radiolysis alone may not be sufficient to result in the structural changes on the timescales observed using SAXS. The presence of additional radical and ionic species, other than H^+^ ions, must therefore play a crucial role in producing the observed rapid changes in SAXS profiles, through mechanisms such as electron or hole catalysis. Despite this, we saw that on increasing the concentration of PS to form higher-concentration LLC mesophases, the X-ray-induced reversal was less pronounced, likely due to the lower water content available for radiolysis.

The X-ray irradiation time required to obtain a good signal-to-noise ratio differs depending on the specific synchrotron beamline or laboratory instrument on which the SAXS experiments are conducted. We have found that the X-ray reversal process can be very fast (<1s), meaning that only a few frames will truly capture the *Z*-isomeric PSS. As the reversal effects are highly sample dependent, varying with photoswitch, concentration, solvent and morphology (e.g., micelles or LLCs), care must be therefore taken when conducting X-ray experiments with photoswitchable materials. As such, a broad-brush approach cannot be applied across all experiments. Instead, we suggest that the *Z-*rich PSS is first irradiated with X-rays for an extended period, whilst taking many short X-ray exposure frames to determine at what point the changes in the scattering pattern become significant. A balance can then be struck between obtaining good-quality data and accurately probing the *Z*-rich PSS for the specific sample and instrument being used. It is only with this additional care that there can be confidence in the structural determination of photoswitchable materials using X-ray radiation. This understanding is vital as the field advances to harness the improved properties of new photoswitches with greater isomerisation control, such as aryliminopyrazoles [46], and to optimise these systems towards applications in solar energy storage, drug delivery or catalysis.

## Experimental

### Materials

Photosurfactants AzoTAB and AAPTAB were synthesised according to previously reported procedures [4,19]. Water was obtained from a Millipore Simak 2 water purification system. Hydrobromic acid (48%) and deuterium oxide (99.9 atom %) were supplied by Merck.

### Sample preparation

To produce micellar samples, light-responsive PS were shaken with the solvent (50 mM in either H_2_O or D_2_O) until homogenous. For samples of higher concentration, AAPTAB was added to water (10 and 90 wt %), and the samples were heated to 60 °C, whilst stirring until homogenous. Samples were left to cool to room temperature whilst stirring.

For the photoisomerisation studies using UV–vis absorbance spectroscopy, samples were irradiated to the PSS in a custom-built LED light box with UV (365 nm, irradiance = 6.00 mW⋅cm^−2^), blue (455 nm, 5.16 mW⋅cm^−2^) or green (525 nm, 0.06 mW⋅cm^−2^) light. For the SAXS studies at high concentration, samples were irradiated in a custom-build LED light box with UV (365 nm) light at an irradiance of 1.24 mW⋅cm^−2^ for 3.5 h. This resulted in 98 ± 2 and 71 ± 4% *E*-to-*Z* isomerisation for 10 and 90 wt % AAPTAB, respectively, as determined previously using ^1^H NMR [20].

### SAXS

SAXS measurements were performed at the high-throughput SAXS beamline B21, Diamond Light Source (Oxfordshire, UK) [47]. The X-ray beam energy was 13.0 keV and the detector distance set to 3.7 m, giving a *Q* range of 0.0045–0.34 Å^−1^.

For the studies at low concentration (50 mM), samples were loaded into a 96 well PCR plate and stored at 25 °C before injection into a quartz capillary and held at 25 °C during measurement. 10 μL of sample was injected into the capillary and held in place during light and X-ray exposure. Frames of 500 ms were taken, and the 2D diffraction patterns were radially averaged and integrated to obtain 1D data. The solvent background was subtracted and frames were averaged using the ScÅtter software [48]. The number of frames averaged was varied to strike a balance between accessing greater time-resolution and achieving a better signal-to-noise ratio. For an X-ray irradiation time of ≤2 s, a single X-ray frame (500 ms) was used, whereas for >2 s, an average of 3 frames was used.

For the measurements at high concentration, samples were loaded into a polyethyleneimine (PEI) capillary (90 wt % water) or using Kapton tape and a 3D-printed lolly stick (10 wt % water). Frames of 250 ms were taken, with a 50 ms wait time between each frame. A total of 900 and 20 frames were taken for the 10 and 90 wt % samples, respectively. The 2D diffraction patterns were radially averaged and integrated to obtain 1D data. The container background was subtracted and sets of three frames were averaged using the ScÅtter software [48].

In-situ light irradiation was achieved using the custom-made setup at beamline B21, using a fibre-coupled pE-4000 (coolLED) focussed onto the sample position and coincident with the X-ray beam [33]. An irradiance of 0.96, 4.00 and 0.42 W⋅m^−2^ was achieved for a wavelength of 365, 460 and 525 nm, as measured before the experiment using a photodiode calibrated to a photothermal power meter.

### Model fitting for SAXS data

SAXS data were fitted using SASFit (version 0.94.11) [34]. The first 50 data points were removed due to aggregation effects in some samples. A linear, horizontal background was set to an appropriate value. The data were fitted to either: ellipsoidal cylindrical core-shell, ellipsoidal core-shell or spherical core-shell structures, with a Gaussian distribution around the (polar) radius to incorporate polydispersity into the model. Data were fitted to the radii (both polar and equatorial for ellipsoidal form factors), shell thickness, scattering length density and length (for cylindrical form factors). The structure factor was fitted using the Hayter–Penfold rescaled mean spherical approximation (RMSA) model to determine the charge and volume fraction of the micelles. Full details of the models used are included in section 3, Supporting Information File 1.

### UV–vis absorbance spectroscopy

UV–vis absorbance spectra were recorded using a Perkin Elmer Lambda 750 spectrometer with a slit width of 2 nm and a scan speed of 266.75 nm⋅min^−1^. Measurements were taken at 1 nm intervals from 700–200 nm, using quartz cuvettes with a 10 mm path length. The temperature was regulated to 25 °C using a Perkin Elmer Peltier temperature controller 201. For the isomerisation experiments using excess acid, HBr (8.9 M, 100 μL) was added to a quartz cuvette containing either AzoTAB (25 μM) or AAPTAB (100 μM) in water in the *Z*-rich PSS. UV–vis absorbance spectra were taken sequentially over 20 cycles every 3 minutes and after leaving the solution overnight in the dark. For experiments over the pH range 5–7, HBr (20 mM) was added to 2 mL of either AzoTAB (100 μM) or AAPTAB (100 μM) in water in the *Z*-rich PSS in a volume of 10 μL for pH 6 and 100 μL for pH 5. UV–vis absorbance spectra were taken sequentially over 66 cycles every 3 minutes.

To investigate the *Z*–*E* isomerisation on X-ray irradiation, UV–vis absorbance spectra were taken using a NanoDrop 1000 spectrometer. Samples (2 μL) were loaded onto the instrument and absorbance from 220 to 750 nm measured.

## Supporting Information

File 1 UV–vis absorbance spectra for photoisomerisation, SAXS using in-situ irradiation, models used for SAXS fitting, micelle dimensions from SAXS fits, calculations for the pH change on X-ray irradiation and UV–vis absorbance spectra for acid-induced isomerisation.

## Data Availability

The data generated and analyzed during this study is openly available in the Apollo data repository at https://doi.org/10.17863/CAM.109905.
