# Supplementary material for: Understanding X-ray-induced isomerisation in photoswitchable surfactant assemblies
Source: Beilstein J Org Chem. 2024 Aug 14;20:2005–15. doi: 10.3762/bjoc.20.176 (PMC11331535; doi:10.3762/bjoc.20.176)
Supplement: File 1 — UV–vis absorbance spectra for photoisomerisation, SAXS using in-situ irradiation, models used for SAXS fitting, micelle dimensions from SAXS fits, calculations for the pH change on X-ray irradiation and UV–vis absorbance spectra for acid-induced isomerisation. [file Beilstein_J_Org_Chem-20-2005-s001.pdf]

BEILSTEIN JOURNAL OF ORGANIC CHEMISTRY

Supporting Information

for

Understanding X-ray-induced isomerisation in photoswitchable surfactant assemblies

Beatrice E. Jones, Camille Blayo, Jake L. Greenfield, Matthew J. Fuchter, Nathan Cowieson and Rachel C. Evans

Beilstein J. Org. Chem. **2024,** *20,* 2005–2015. doi:10.3762/bjoc.20.176

UV–vis absorbance spectra for photoisomerisation, SAXS using in-situ irradiation, models used for SAXS fitting, micelle dimensions from SAXS fits, calculations for the pH change on X-ray irradiation and UV–vis absorbance spectra for acid-induced isomerisation

License and Terms: This is a supporting information file under the terms of the Creative Commons Attribution License (https://creativecommons.org/licenses/by/4.0). Please note that the reuse, redistribution and reproduction in particular requires that the author(s) and source are credited and that individual graphics may be subject to special legal provisions.
The license is subject to the *Beilstein Journal of Organic Chemistry* terms and conditions: (https://www.beilstein-journals.org/bjoc/terms)

TABLE OF CONTENTS

1 UV–VIS ABSORBANCE SPECTRA FOR PHOTOISOMERISATION S2

2 SMALL-ANGLE X-RAY SCATTERING USING IN-SITU IRRADIATION S2

3 MODELS USED FOR SAXS FITTING S4

4 MICELLE DIMENSIONS FROM SAXS FITS S7

5 UV-VIS ABSORBANCE SPECTRA BEFORE AND AFTER X-RAY IRRADIATION S10

6 CALCULATIONS FOR THE PH CHANGE ON X-RAY IRRADIATION S11

7 CALCULATIONS FOR TEMPERATURE CHANGE ON X-RAY IRRADIATION S12

8 UV–VIS ABSORBANCE SPECTRA FOR ACID-INDUCED ISOMERISATION S12

9 REFERENCES S13

1 UV–vis absorbance spectra for photoisomerisation

On irradiation with UV (365 nm) light, a change in the UV–vis absorbance spectrum indicates photoisomerisation for both AzoTAB and AAPTAB to form a photostationary state (PSS) of mostly *Z* isomers (Figure S1). On subsequent irradiation with blue light, AzoTAB shows high reversibility into the *E* isomeric form. In contrast, the response of AAPTAB is much slower, showing only minimal change over a 20-minute irradiation period with green light. The change in peak intensity for AzoTAB in the *E*-rich state in the native form and blue PSS is thought to be due to a difference in the dissolved concentration of the surfactant before and after light irradiation.

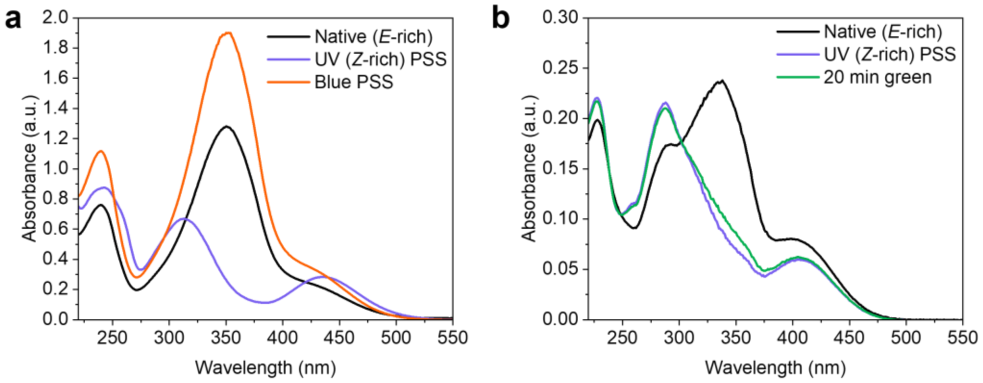

Figure S1: UV–vis absorbance spectra of (a) AzoTAB (50 μM in water) and (b) AAPTAB (100 μM in water) in the native state, which consists of mostly the *E* isomer, and after irradiation with UV (365 nm) light for 15 minutes to form a PSS of mostly *Z* isomers. Subsequent irradiation with blue (460 nm) triggers *Z*–*E* isomerisation in AzoTAB. In AAPTAB, irradiation with green (525 nm) light for 20 minutes shows only a small change in the absorbance spectrum.

2 Small-angle X-ray scattering using in-situ irradiation

For AzoTAB, on irradiation of the *Z*-PSS with in-situ blue (460 nm) irradiation, the SAXS signal shows a gradual return towards the original pattern given by the *E* isomer (Figure S2a). Data fits indicate that this is associated with a return to the ellipsoidal cylindrical micelle morphology, with polar and equatorial radii of 25 and 14 Å, and a length of 104 Å, which is shorter than the 136 Å in the original state (Table S1). This discrepancy in the micelle size on reverse isomerisation could be due to slow agglomeration of the micelles into longer cylinders over time. Evidence of larger-scale aggregates in the *E* isomer for this sample, before UV or blue irradiation is visible as a straight-line power-law decay in the low-*Q* region of the SAXS curve (Figure S2a). The decay follows the relationship $I(Q) \propto Q^{-2}$, which is characteristic of random-walk interactions from worm-like micelles[1,2]. This suggests that there is heterogeneity in some samples, leading to larger-scale cylindrical forms that begin to behave akin to worm-like micelles. *Z*–*E* isomerisation in azobenzene photoswitches can also be induced using gentle heating due to the low activation energy for the reversal to the thermodynamically stable, *Z* state[3]. On heating AzoTAB in the *Z*-rich PSS to 55 °C and holding it in the dark, a return of the SAXS profile towards that of the original, *E* isomer was observed (Figure S2b), as expected due to thermally-induced reverse isomerisation at these temperatures.

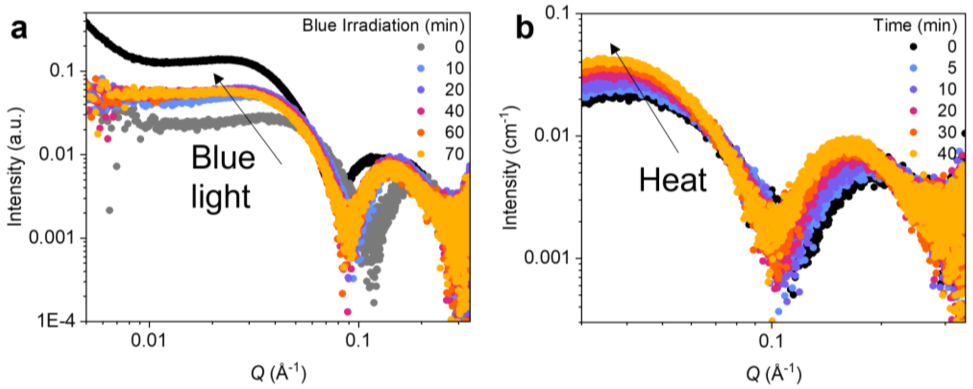

Figure S2: SAXS patterns showing the effects of in-situ (a) blue light irradiation (460 nm) and (b) heating at 55 °C on the self-assembly of AzoTAB (50 mM in water) in the *Z*-rich photostationary state. Note that the original, unirradiated sample in (a) is given by the black circles.

For AAPTAB in the Z-rich PSS, on irradiation with green light there is little change in the SAXS signal (Figure S3). On heating to 55 °C, the Guinier plateau region remains unchanged, which indicates little change in the micelle shape and size; however, there is decrease in the gradient of the straight-line power-law decay scattering in the low-*Q* region, indicating a loss of large-scale aggregates in the solution which could be forming due to a lower solubility of the AAPTAB at room temperature.

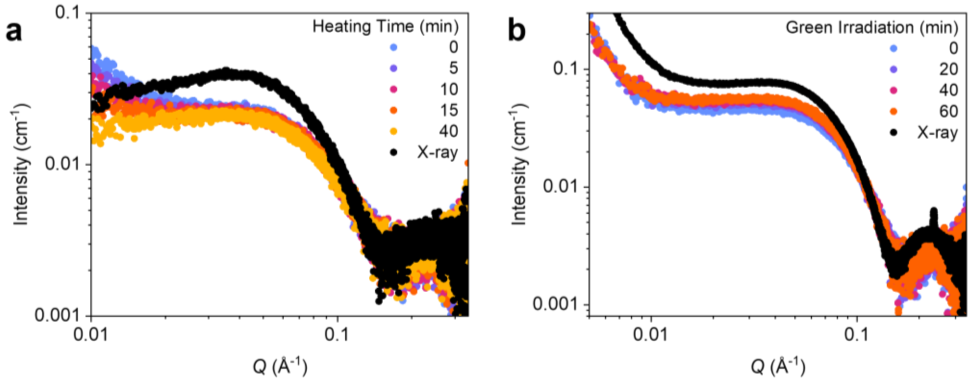

Figure S3: SAXS patterns showing the effects of in-situ (a) heating to 55 °C and (b) irradiation with green (525 nm) light for AAPTAB (50 mM in water) on showing minimal change over 40–60 minutes. In comparison, irradiation with 50 s of X-rays (black) indicates a dramatic change in the structure.

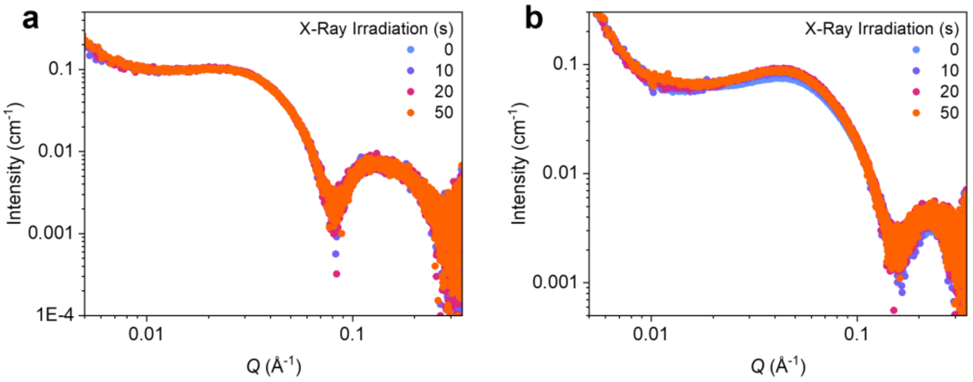

Figure S4: SAXS patterns showing the negligible effects of X-ray exposure time on (a) AzoTAB and (b) AAPTAB (both 50 mM in water) in the native, *E* isomer.

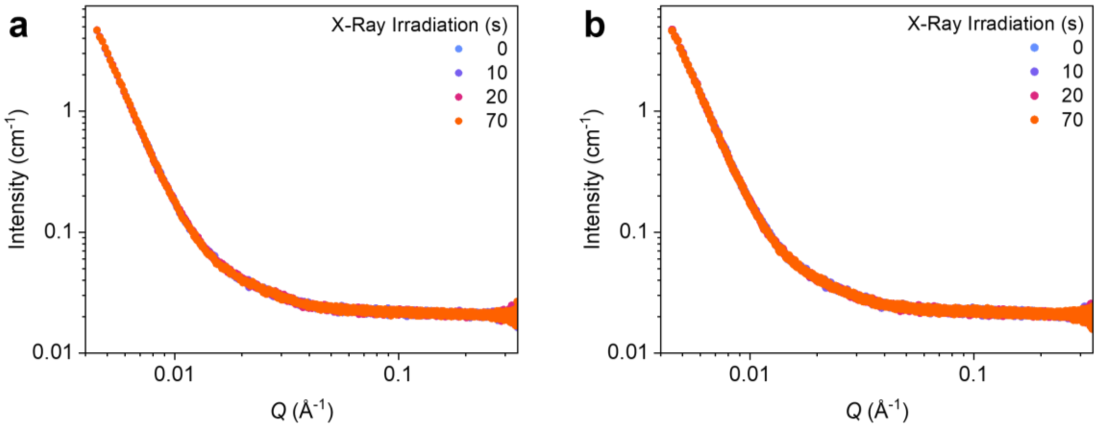

Figure S5: SAXS patterns show no change in the backgrounds of (a) H_2O and (b) D_2O on X-ray irradiation of up to 70 s.

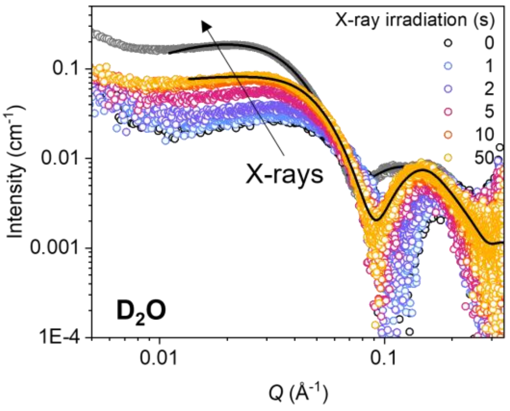

Figure S6: SAXS curves for AzoTAB (50 mM) in deuterium dioxide (D_2O) showing the changes in form factor, and therefore micelle size and shape, after UV (365 nm) irradiation to the *Z*-rich PSS and subsequent X-ray irradiation. The solid black lines indicate the results from model fitting to the SAXS data. The grey circles indicate the original, *E* isomeric state.

3 Models used for SAXS Fitting

The SAXS scattering profiles were modelled in SASfit software (version 0.94.11)[4]. This minimises the goodness-of-fit, X_r^2, by adjusting the model parameters, to match the model intensity with the measured intensity. Goodness-of-fit, X_r^2 is defined as:

$$X_r{}^2 = \frac{1}{(N-M)}\sum_{i=1}^{N}\left[\frac{I_{exp}(q_i) - I_{mod}(q_i)}{\sigma_{exp}(q_i)}\right]^2 \qquad \text{Eq. S1}$$

where $I_{exp}(q)$ and $I_{mod}(q)$ are the measured and modelled intensities, respectively, and $\sigma_{exp}(q)$ is the uncertainty on the intensity.

The model intensity, $I_{mod}(q)$ is defined as:

$$I_{mod}(q) = \sum_{i=1}^{N} \left\{ \left[\int_a^b P(q,x) f(x)\, \mathrm{d}x \right] S(q) \right\} \qquad \text{Eq. S2}$$

where $P(q, x)$ is the form factor, describing the shape of the scatterer, $f(x)$ is a shape parameter distribution, and $S(q)$ is the structure factor, describing interactions between the scatterers.

The form factor for the elliptical cylindrical shell in SASfit, $I_{Shell}(q)$ is defined by the following equation:

$$I_{Shell}(q, R_1, R_2, \Delta\eta, \mu) = [K(q, R_1, \Delta\eta) - K(q, R_2, \Delta\eta(1\text{-}\mu))]^{\,2} \qquad \text{Eq. S3}$$

with

$$K(q, R, \Delta\eta) = \frac{4}{3}\pi R^3 \Delta\eta\, 3\, \frac{\sin QR - QR\cos QR}{(QR)^3} \qquad \text{Eq. S4}$$

The forward scattering for $q = 0$ is given by:

$$\lim_{q=0} I_{Shell}(q, R_1, R_2, \Delta\eta, \mu) = \left(\frac{4}{3}\pi\Delta\eta\ [R_1^3 - R_2^3(1-\mu)]\right)^2 \qquad \text{Eq. S5}$$

where R_1 = overall radius of the spherical shell

R_2 = radius of spherical core

$\Delta\eta$ = scattering length density difference between the shell and the solvent

μ = scattering length density difference between the core and the matrix relative to the shell

The form factor for the ellipsoid of revolution in SASfit, $I_{ECSh}(q)$ is defined by the following equation:

$$I_{ECSh}(q) = \int_0^1 [F(q, R_p, R_{eq}, t, \mu)]^{\,2} \mathrm{d}\mu \qquad \text{Eq. S6}$$

with

$$F(q, R_p, R_{eq}, t, \mu) = (\eta_{core}\text{-}\ \eta_{shell}) V_c \left[\frac{3 j_1(x_c)}{x_c}\right] + (\eta_{shell}\text{-}\ \eta_{sol}) V_t \left[\frac{3 j_1(x_t)}{x_t}\right] \qquad \text{Eq. S7}$$

$$j_1(x) = \frac{\sin(x) - x cos(x)}{x^2}$$

$$x_c = Q\sqrt{R_p^2\,\mu^2 + R_e^2(1-\mu^2)}$$

$$x_t = Q\sqrt{(R_p + t)^2\mu^2 + (R_e + t)^2(1 - \mu^2)}$$

$$V_c = \frac{4}{3}\pi R_p R_e^2$$

$$V_t = \frac{4}{3}\pi(R_p + t)(R_e + t)^2$$

where η_{core} = scattering length density of core

η_{shell} = scattering length density of shell

η_{sol} = scattering length density of solvent

R_P = polar semi-axis of elliptical core

R_e = equatorial semi-axis of elliptical core

t = thickness of shell

V_c = volume of core

V_t = total volume of core along with shell

The form factor for a randomly oriented cylindrical shell with elliptical cross-section in SASfit, $I_{ellCyl}(q)$ is defined by the following equation:

$$K_{ellCyl}(q, \Delta\eta, R, \varepsilon, L, t, \varphi, \alpha) = \pi\varepsilon R(\varepsilon R + t)L\Delta\eta \times \frac{2J_1(qr(R, \varepsilon, t, \varphi, \alpha))}{qr(R, \varepsilon, t, \varphi, \alpha)} \frac{\sin(q\frac{L}{2}\cos(\alpha))}{q\frac{L}{2}\cos(\alpha)} \quad \text{Eq. S8}$$

$$r(R, \varepsilon, t, \varphi, \alpha) = \sqrt{(R + t)^2\sin^2(\varphi) + (\varepsilon R + t)^2\cos^2(\varphi)} \quad \text{Eq. S9}$$

$$I_{ellCyl}(q) = \frac{2}{\pi}\iint_0^{\frac{\pi}{2}} (K_{ellCyl}(q, \eta_{core} - \eta_{shell}, R, \varepsilon, L, t, \varphi, \alpha) + K_{ellCyl}(q, \eta_{shell} - \eta_{sol}, R, \varepsilon, L, t, \varphi, \alpha))^2 \sin(\alpha)d\alpha\, d\varphi \quad \text{Eq. S10}$$

where R = radius of the micelle core

ε = stretching factor of ellipsoid

L = cylinder length

T = shell thickness

η_{core} = scattering length density of cylinder core

η_{shell} = scattering length density of cylinder shell

η_{sol} = scattering length density of solvent

4 Micelle Dimensions from SAXS Fits

Table S1: Fitted parameters from modelling of the SAXS data for AzoTAB (50 mM) in water and D_2O after varying UV, X-ray and blue light irradiation times, where R_p is the polar radius, R_{eq} is the equatorial radius, t is the shell thickness, L is the cylinder length, SLD is the scattering length density, Z is the micelle charge, η is the micelle volume fraction and χ^2 is the goodness-of-fit.

Irradiation time			Model	R_p / Å	R_{eq} / Å	t / Å	L / Å	SLD		Z	η	X^2
UV (min)	X-ray (s)	Blue (min)						Core	Shell			
H_2O												
0	0	0	Elliptical cylindrical shell	31.39 ± 0.05	11.46 ± 0.04	13.28 ± 0.02	136.0 ± 0.2	5.1×10^{-6}	1.84×10^{-5}	16.9	0.08	3.56
80	0	0	Ellipsoidal shell	13.25 ± 0.14	19.32 ± 0.20	10.25 ± 0.18		6.0×10^{-6}	1.36×10^{-5}	11.1	0.09	0.85
80	50	0	Elliptical cylindrical shell	12.69 ± 0.12	14.67 ± 0.14	7.77 ± 0.11	97.5 ± 0.6	5.0×10^{-6}	2.8×10^{-5}	11.0	0.08	0.68
80	1.5	70	Elliptical cylindrical shell	25.01 ± 0.15	13.78 ± 0.18	8.80 ± 0.13	103.5 ± 1.1	4.9×10^{-6}	2.2×10^{-5}	11.0	0.08	0.74
D_2O												
0	0	0	Elliptical cylindrical shell	34.86 ± 0.05	12.17 ± 0.05	12.77 ± 0.04	201 ± 1	5.9×10^{-6}	1.55×10^{-5}	15.6	0.10	4.19
80	0	0	Ellipsoidal shell	15.4 ± 0.1	23.3 ± 0.3	4.4 ± 0.4		6.0×10^{-6}	1.9×10^{-5}	11.4	0.07	0.82
80	50	0	Elliptical cylindrical shell	24.1 ± 0.1	12.8 ± 0.2	11.1 ± 0.1	106 ± 1.2	4.3×10^{-6}	1.3×10^{-5}	6.8	0.08	0.87

Table S2: Fitted parameters from modelling of the SAXS data for AAPTAB (50 mM) in water after varying UV, X-ray and blue light irradiation times, where R_p is the polar radius, R_{eq} is the equatorial radius, t is the shell thickness, SLD is the scattering length density, Z is the micelle charge, η is the micelle volume fraction and χ^2 is the goodness-of-fit.

**Irradiation time**		**Model**	**R_p / Å**	**R_{eq} / Å**	**t / Å**	**SLD**		**Z**	**η**	**X^2**
**UV (min)**	**X-ray (s)**					**Core**	**Shell**			
					H_2O					
0	0	Ellipsoidal shell	23.8 ± 0.1	13.41 ± 0.03	7.07 ± 0.06	9.0×10^{-6}	1.9×10^{-5}	3	0.11	3.63
10	0	Ellipsoidal shell	25.1 ± 0.2	13.28 ± 0.03	5.42 ± 0.01	9.0×10^{-6}	2.2×10^{-5}	2.7	0.10	0.80
20	0	Ellipsoidal shell	22.3 ± 0.4	13.07 ± 0.14	5.16 ± 0.30	9.0×10^{-6}	2.2×10^{-5}	0.44	0.09	1.01
30	0	Ellipsoidal shell	23.9 ± 0.2	13.49 ± 0.05	3.69 ± 0.01	9.1×10^{-6}	2.6×10^{-5}	0.44	0.09	1.03
40	0	Spherical shell	19.1 ± 0.1		5.6 ± 0.2	9.4×10^{-6}	2.0×10^{-5}	3.82	0.07	0.96
50	0	Spherical shell	18.7 ± 0.1		5.2 ± 0.2	9.4×10^{-6}	2.0×10^{-5}	4.33	0.07	0.97
60	0	Spherical shell	19.0 ± 0.1		6.4 ±0,1	9.4×10^{-6}	1.9×10^{-5}	2.68	0.07	0.72
70	0	Spherical shell	18.5 ± 0.1		5.1 ± 0.3	9.4×10^{-6}	2.0×10^{-5}	3.32	0.07	0.99
80	0	Spherical shell	18.4 ± 0.1		5.6 ± 0.3	8.4×10^{-6}	1.9×10^{-5}	3.38	0.07	0.88
80	0.5	Spherical shell	19.2 ± 0.2		5.8 ± 0.2	9.4×10^{-6}	1.8×10^{-5}	3.78	0.07	0.58
80	1	Spherical shell	19.5 ± 0.2		6.6 ± 0.2	9.4×10^{-6}	1.8×10^{-5}	4.04	0.07	0.62
80	2	Spherical shell	19.9 ± 0.2		6.7 ± 0.2	9.3×10^{-6}	1.8×10^{-5}	4.41	0.07	0.66
80	5	Ellipsoidal shell	21.2 ± 0.3	12.5 ± 0.1	7.0 ± 0.2	9.0×10^{-6}	1.9×10^{-5}	0.38	0.09	0.95
80	10	Ellipsoidal shell	20.2 ± 0.3	12.0 ± 0.1	9.4 ± 0.2	9.0×10^{-6}	1.7×10^{-5}	0.11	0.10	0.90
80	50	Ellipsoidal shell	23.8 ± 0.2	13.4 ± 0.1	6.8 ± 0.2	9.0×10^{-6}	1.9×10^{-5}	2.79	0.10	0.86

Table S3: Fitted parameters from modelling of the SAXS data for AzoTAB (50 mM) in D_2O after varying UV, X-ray and blue light irradiation times, where R_p is the polar radius, R_{eq} is the equatorial radius, t is the shell thickness, SLD is the scattering length density, Z is the micelle charge, η is the micelle volume fraction and χ^2 is the goodness-of-fit.

Irradiation time		Model	R_p / Å	R_{eq} / Å	t / Å	SLD		Z	η	X^2
UV (min)	X-ray (s)					Core	Shell			
				D_2O						
0	0	Ellipsoidal shell	23.4 ± 0.1	11.49 ± 0.04	11.7 ± 0.1	9.0×10^{-6}	1.4×10^{-5}	7.44	0.06	1.67
10	0	Ellipsoidal shell	26.2 ±1.2	14.7 ± 0.6	4.6 ± 1.1	8.5×10^{-6}	1.7×10^{-5}	1	0.04	0.51
20	0	Ellipsoidal shell	21.5 ± 0.8	11.0 ± 0.3	10.0 ± 0.5	8.5×10^{-6}	1.2×10^{-5}	1	0.03	0.52
40	0	Ellipsoidal shell	20.0 ± 0.3	20.0 ± 0.3	5.6 ± 0.2	1.0×10^{-7}	6.1×10^{-6}	1	0.02	0.62
60	0	Spherical shell	19.9 ± 0.3		5.4 ±0.2	1.0×10^{-7}	6.2×10^{-6}	1	0.02	0.62
80	0	Spherical shell	21.4 ± 0.2		9.0 ± 0.5	8.5×10^{-6}	4.3×10^{-6}			0.72
80	0.5	Spherical shell	20.4 ± 0.4		6.9 ± 0.5	1.0×10^{-7}	5.2×10^{-6}			0.59
80	1	Spherical shell	20.7 ± 0.4		7.5 ± 0.4	1.0×10^{-7}	5.1×10^{-6}			0.57
80	2	Spherical shell	20.4 ± 0.4		6.5 ± 0.4	1.0×10^{-7}	5.8×10^{-6}			0.61
80	5	Spherical shell	20.9 ± 0.2		5.8 ± 0.2	1.0×10^{-7}	6.4×10^{-6}	1.06	0.03	0.74
80	10	Spherical shell	21.9 ± 0.2		6.9 ± 0.2	1.0×10^{-7}	5.8×10^{-6}	1.00	0.03	0.67
80	50	Spherical shell	24.5 ± 0.1		10.9 ± 0.3	9.0×10^{-6}	5.0×10^{-6}	1.15	0.03	0.68

5 UV-Vis absorbance spectra before and after X-ray irradiation

To investigate the *Z*–*E* isomerisation of AAPTAB during X-ray irradiation, UV-Vis absorbance spectroscopy was used. AAPTAB (1 mM in water) was irradiated to form the Z isomer (Figure S7). This was then loaded into the beamline, irradiated with X-rays (1 s), and re-cuperated. The UV-Vis absorbance spectrum after irradiation shows some changes (Figure S7b), which indicate a percentage of the sample has reversed back to the *E* isomer. However, the observed change is small due to the small volume of sample that is irradiated by the X-ray beam (0.375 μL) in comparison to the volume of the sample (10 μL) that has been re-cuperated to run the UV-Vis spectrum. The volume loaded is the lower limit that can be used so that the sample stays centred on the X-ray beam during measurement, so this could not be modified further. However, due to the high stability of the *Z* isomer for AAPTAB, we can assume that this small contribution of *Z*–*E* isomerisation has been induced by the X-ray beam, rather than thermal isomerisation during the time taken for the measurements. We note that the UV-Vis absorbance spectra here are taken on a NanoDrop 1000 spectrophotometer, and therefore more quantitative results cannot be obtained, as this was all that was available at the synchrotron facility.

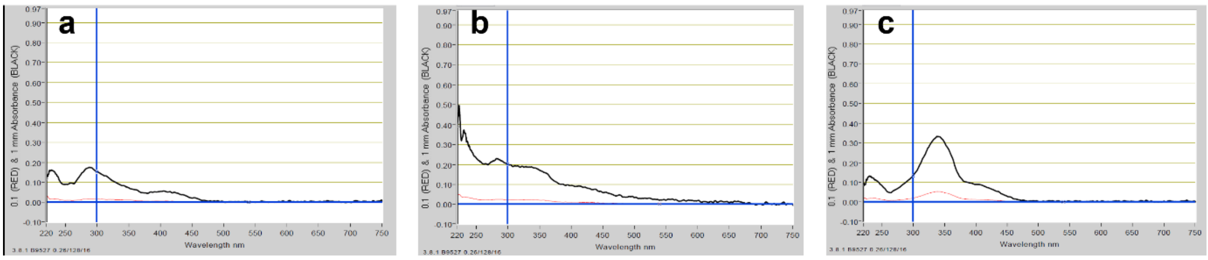

Figure S7: UV-Vis absorbance spectra for AAPTAB (1 mM in water) (a) in the *Z*-rich PSS, before X-ray irradiation, and (b) after 1 s of X-ray irradiation, showing partial reversion back to the spectrum given by (c) native, E isomer. Note that the spectra in red correspond to 0.1 mm absorbance and in black to 1 mm absorbance

6 Calculations for the pH change on X-ray irradiation

To estimate the pH change on X-ray irradiation for the X-ray flux at beamline B21, Diamond Light Source, the absorbed X-ray dose rate, *D*, was first calculated using the relationship[5]:

$$D = \frac{E_T}{m} \quad \text{Eq. S11}$$

$$D = \frac{I_0 \times E \times t\ (1 - \exp(-\mu_{ic}\,(E) \times\ \lambda\,))}{A \times\ \lambda\ \times \rho}$$

where, E_T/m is the transferred energy per mass, m, I_0 is the incident X-ray beam intensity, E is the X-ray energy, *t* is the exposure time, μ_{ic} is the incoherent contribution of the attenuation coefficient, A is the beam area, ρ is the beam density and λ is the beam attenuation length. The parameters used and source of the information are summarised in Table S4.

Table S4: Parameters used for the calculation of the absorbed X-ray dose rate for the experimental conditions used at beamline B21, Diamond Light Source, and the source of the parameter values.

Parameter	Description	Value	Source
I_0	Beam Intensity	4 x 10^{12} photons s^{-1}	From experiment
E	X-ray energy	13 keV	From experiment
t	Exposure time	500 ms	From experiment
μ_{ic}	Incoherent contribution of the attenuation coefficient	2.325 cm^2 g^{-1}	Calculated for H_2O at 13 keV [6]
A	Beam area	1.0 x 0.25 mm	From experiment
λ	Attenuation length	0.403 cm or 0.402 g cm^{-2}	Calculated for H_2O at 13 keV [6]
ρ	Density	997 kg m^{-3}	Density of water

The resulting dose is *D* = 1.6 kGy (note that 1 Gy = 1 J kg^{-1}) for a single X-ray frame of 500 ms or a dose rate of $\dot{D}$ = 3.2 kGy s^{-1}. Fritsch et al. used kinetic modelling to simulate the acid-base chemistry of neat, aerated water as a function of dose rate of incident X-ray radiation and the initial pH value of the solution[7]. By comparing their results to the X-ray dose rate calculated here, ≈10^3 Gy s^{-1}, we can expect a solution of pH 7 to change to pH 5–6. It is worth noting that the addition of free bromide ions will also have an effect on this pH change. For additions of between 1 and 10 mM of Br^-, Fritsch et al. calculated that the pH change for an X-ray dose rate of ≈10^3 Gy s^{-1} varies between 5.6 and 5.7[7]. We therefore chose to investigate the effect of pH 5 and 6 on isomerisation properties in this work. Hydrobromic acid was used to modify the pH as the Br^- counterion is identical to the photosurfactant counterions, minimising additional chemical effects on the system.

7 Calculations for temperature change on X-ray irradiation

To estimate the temperature change induced at the sample position due to the X-ray beam, we can use the absorbed X-ray dose rate calculated above, $\dot{D}$ = 3.2 kGy s^{-1}, or 3.2 J kg^{-1} (Section 6, Equation S11). If we adopt an upper-limit scenario where all this energy is transferred to heat energy, we can calculate the induced temperature change in the water using the equation[8]:

$$Q = m\, c\, \Delta T \qquad \text{Eq. S12}$$

where Q is the heat energy transferred, m is the mass of water, c is the specific heat capacity of water (4180 J kg^{-1} K^{-1}[8]) and ΔT is the temperature change. This gives a temperature change of 0.38 °C over a single, 500 ms X-ray frame, or 38 °C over the longest X-ray exposure of 50 s. However, these calculations assume that there is no heat transfer to the surrounding sample or cooling system. This cooling system actively keeps the sample capillary at 25 °C during measurement, meaning these calculations will be an over-estimate, especially at longer irradiation times.

Taking this upper-limit scenario, the amount of *Z*–*E* isomerisation induced for 50 s X-ray exposure would be ~2 % for AzoTAB, which shows the greatest thermal instability in comparison to AAPTAB. This is calculated by taking the thermal rate constant at 60 °C from an Arrhenius plot for a similar, AzoTAB surfactant [9]. The AAPTAB will be even more stable to heating, meaning that the effect on this molecule can be assumed to be negligible under these conditions. Using these calculations, we can conclude that thermal effects from the X-ray beam will be minimal in modifying the ratio of *E* to *Z* isomers in these systems.

8 UV–vis absorbance spectra for acid-induced isomerisation

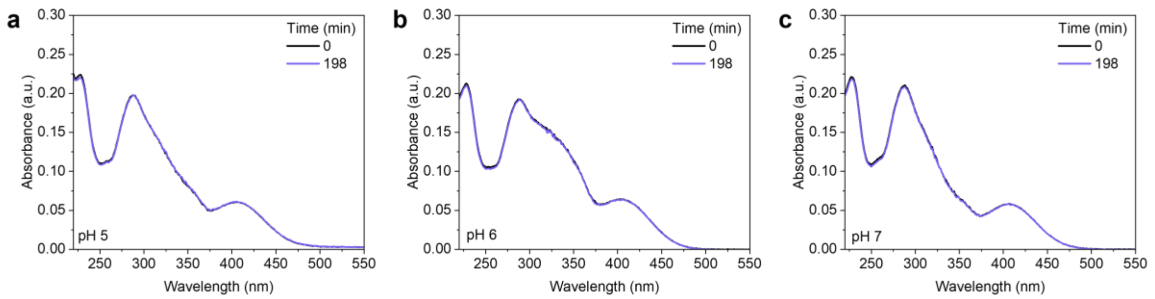

Figure S8: UV–vis absorbance spectra for AAPTAB (100 µM in water) in the *Z*-rich PSS (obtained after UV, 365 nm, irradiation for 15 minutes) as a function of time held at 25 °C in the dark on addition of hydrobromic acid to form a pH of (a) 5, (b) 6 and (c) 7.

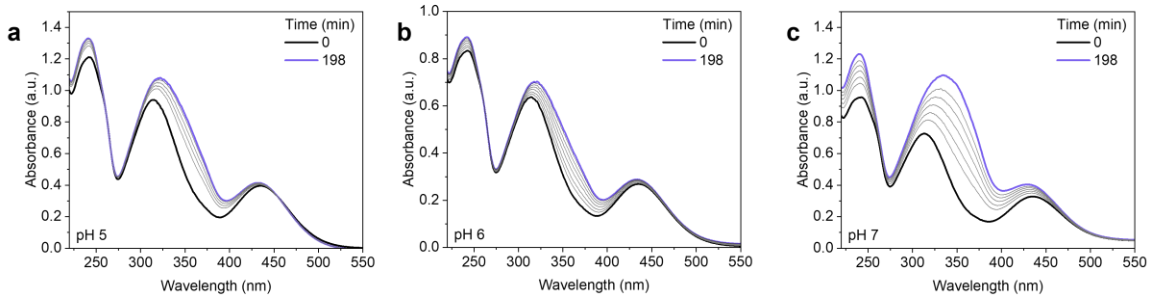

Figure S9: UV–vis absorbance spectra for AzoTAB (100 μM in water) in the *Z*-rich PSS (obtained after UV, 365 nm, irradiation for 15 minutes) as a function of time held at 25 °C in the dark on addition of hydrobromic acid to form a pH of (a) 5, (b) 6 and (c) 7. The grey lines indicate the spectrum every 30 minutes between the first and final timestamps.

9 References

(1) Dreiss, C. A. *Soft Matter* **2007**, *3*, 956–970. doi:10.1039/b705775j

(2) Pedersen, J. S.; Schurtenberger, P. *Scattering Functions of Semiflexible Polymers with and without Excluded Volume Effects*; 1996

(3) Bandara, H. M. D.; Burdette, S. C. *Chem Soc Rev* **2012**, *41*, 1809–1825. doi:10.1039/c1cs15179g

(4) Breßler, I.; Kohlbrecher, J.; Thünemann, A. F. *J Appl Crystallogr* **2015**, *48*, 1587–1598. doi:10.1107/S1600576715016544

(5) Ober, M. F.; Müller-Deku, A.; Baptist, A.; Ajanović, B.; Amenitsch, H.; Thorn-Seshold, O.; Nickel, B. *Nanophotonics* **2022**, *11*, 2361–2368. doi:10.1515/nanoph-2022-0053

(6) Berger, M. J.; Hubbell, J. H.; Seltzer, S. M.; Chang, J.; Coursey, J. S.; Sukumar, R.; Zucker, D. S.; Olsen, K. XCOM: Photon Cross Sections Database

(7) Fritsch, B.; Körner, A.; Couasnon, T.; Blukis, R.; Taherkhani, M.; Benning, L. G.; Jank, M. P. M.; Spiecker, E.; Hutzler, A. *J Phys Chem Lett* **2023**, *14*, 4644–4651. doi:10.1021/acs.jpclett.3c00593

(8) Almousa, N. H; Alotaibi, M. R.; Alsohybani, M.; Radziszewski, D.; AlNoman, S. M.; Alotaibi, B. M.; Khayyat, M. M. *Crystals* **2021**, *11* (8), 951. doi: 10.3390/cryst11080951

(9) Arya, P.; Jelken, J.; Lomadze, N.; Santler, S.; Bekir, M. *J. Chem. Phys*. **2020**, *152,* 024904. doi: 10.1063/1.5135913
